# [Corrigendum] The interaction between miR-148a and DNMT1 suppresses cell migration and invasion by reactivating tumor suppressor genes in pancreatic cancer

**DOI:** 10.3892/or.2026.9195

**Published:** 2026-09-16

**Authors:** Le Hong, Gen Sun, Long Peng, Yi Tu, Zhen Wan, Haiwei Xiong, Yong Li, Weidong Xiao

Oncol Rep 40: 2916–2925, 2018; DOI: 10.3892/or.2018.6700

Subsequently to the publication of the above article, an interested reader drew to the Editor's attention that, in Fig. 2C and D on p. 2921 showing the methylation status of the miR-148a promoter, the centrally placed ‘M’ and ‘U’ lanes in these figure parts apparently showed the same data, albeit with horizonal stretching of the bands in question. Furthermore, in Fig. 6B on p. 2923, which showed the results of cell migration and invasion assay experiments, the ‘Mimics-NC/Migration’ and ‘Blank/Invasion’ data panels apparently showed the same data, albeit with different sizing of the panels, such that these were derived from the same orginal source. Finally, the reader noted that one of the antibodies the authors had used in their study was apparently selected inappropriately: The antibody (cat. no. ab51243) that the authors reported to have used is specific for the protein known as p16-ARC (or ARPC5), not the apoptosis-associated protein p16-INK4a, as was intended.

The authors were asked to investigate these various matters, and have fully responded to the reader's queries. Concerning the antibody, after thoroughly examining the original reagent purchase records and raw experimental data, the authors can confirm that the correct anti-CDKN2A/p16 antibody (cat. no. ab108349; Abcam) was in fact used in this study: As the catalogue number of antibody was not stated in the initial manuscript submission and this information was requested during the editing stage of the paper, the authors inadvertently entered the incorrect catalogue number into the text of the paper (they also provided a copy of the original purchase form to the Editorial Office for our inspection). Therefore, the sentence commencing on p. 2918, left-hand column, line 20, in the ‘*Protein extraction and western blot analysis*’ subsection of the Materials and methods section should have read as follows: ‘The primary antibodies used were as follows: DNMT1 (1:500, cat. no. ab188453; Abcam), p16 (1:200, cat. no. ab108349; Abcam)...’.

Concerning Figs. 2 and 6, the authors were able to examine their original data, and realized that errors had inadvertently been made when assembling these figures. Corrected versions of Figs. 2 and 6, now showing data from one of the repeated sets of experiments for Fig. 2A-D and the correct data for the ‘Blank/Invasion’ data panel in Fig. 6B, are shown on the next two pages. The authors wish to emphasize that the errors made in assembling the data in these figures did not affect the overall conclusions reported in the paper. The authors are grateful to the Editor of *Oncology Reports* for granting them this opportunity to publish a Corrigendum, and apologize to both the Editor and the readership for any inconvenience caused; they also thank the reader of the article for drawing these matters to their attention.

## Figures and Tables

**Figure 2.**
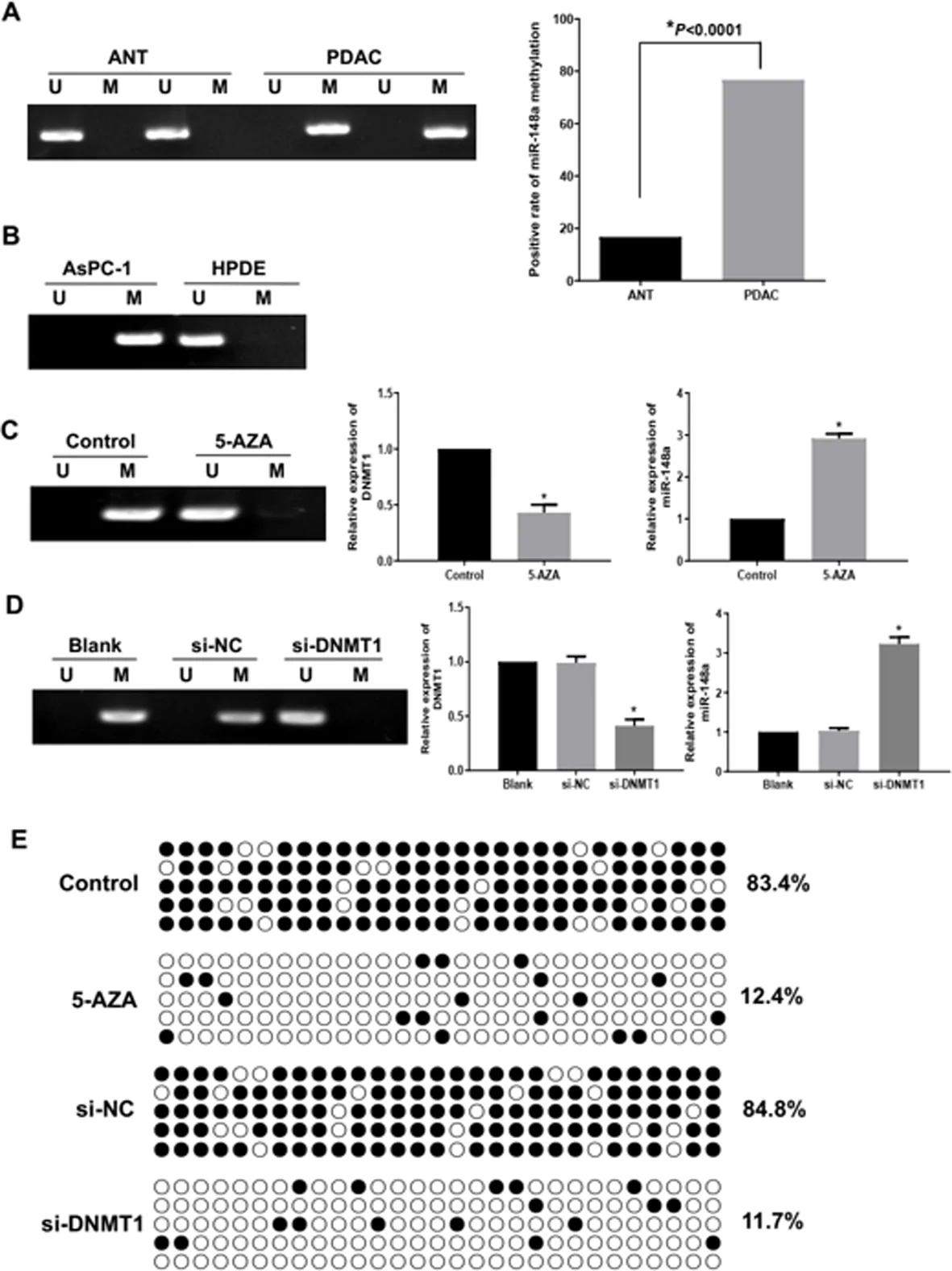
Methylation status of the miR-148a promoter. (A) Methylation status of the miR-148a promoter in ANT and PDAC specimens. (B) Methylation status of the miR-148a promoter in HPDE and AsPC-1 cells. (C) Effects of 5-Aza-CdR on methylation status of the miR-148a promoter, and DNMT1 and miR-148a expression (*P<0.01). (D) Effects of si-DNMT1 on methylation status of the miR-148a promoter, and DNMT1 and miR-148a expression (*P<0.01). (E) Bisulfite sequencing polymerase chain reaction analysis of CpG sites in the miR-148a promoter in AsPC-1 cells with or without 5-Aza-CdR treatment and si-DNMT1 transfection. Open and filled circles represent U and M CpG sites, respectively. Each horizontal row represents a single clone. There are 29 CpG sites. 5-AZA, 5-Aza-2’-deoxycytidine; ANT, adjacent non-tumorous; DNMT1, DNA methyltransferase 1; M, methylated; miR-148a, microRNA-148a; NC, negative control; PDAC, pancreatic ductal adenocarcinoma; si, small interfering RNA; U, unmethylated.

**Figure 6.**
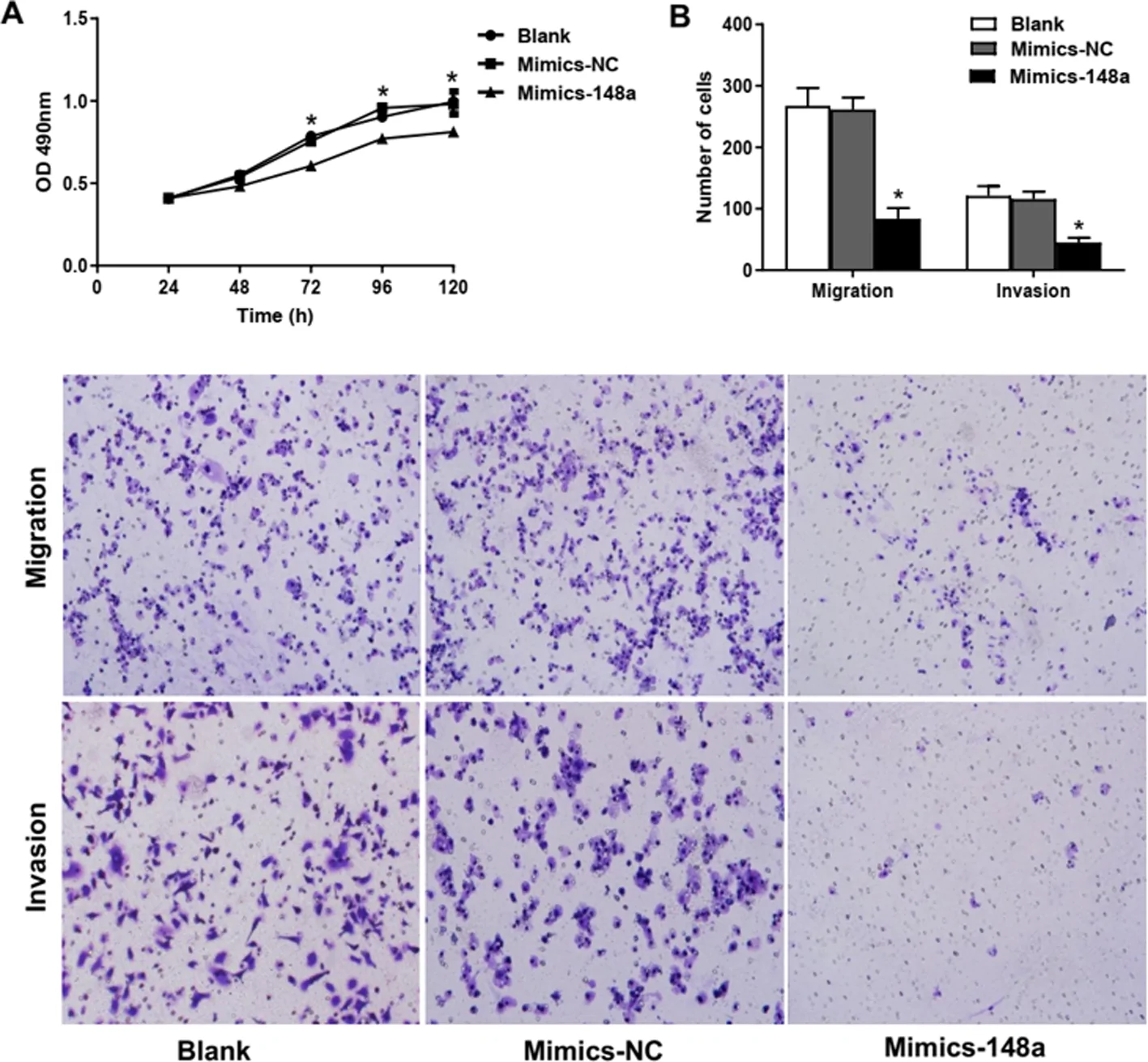
miR-148a inhibits proliferation, migration and invasion of AsPC-1 cells in vitro. (A) Cell proliferation was determined in AsPC-1 cells post-transfection with miR-148a mimics or mimics-NC. *P<0.05 compared with the blank group and mimics-NC group. (B) Migration and invasion were determined in AsPC-1 cells post-transfection with miR-148a mimics or mimics-NC (magnification, ×100). *P<0.01 compared with the blank group and mimics-NC group. miR-148a, microRNA-148a; NC, negative control; OD, optical density.

